# Identification of Early Clinical and Histological Factors Predictive of Kasai Portoenterostomy Failure

**DOI:** 10.3390/jcm11216523

**Published:** 2022-11-03

**Authors:** Caroline P. Lemoine, Hector Melin-Aldana, Katherine A. Brandt, Riccardo Superina

**Affiliations:** 1 Division of Transplant and Advanced Hepatobiliary Surgery, Ann & Robert H. Lurie Children’s Hospital of Chicago, Northwestern University Feinberg School of Medicine, Chicago, IL 60611, USA; 2 Department of Pathology, Ann & Robert H. Lurie Children’s Hospital of Chicago, Northwestern University Feinberg School of Medicine, Chicago, IL 60611, USA

**Keywords:** biliary atresia, Kasai portoenterostomy, liver histology, transplant-free survival

## Abstract

Background: It is impossible to predict which patients with biliary atresia (BA) will fail after Kasai portoenterostomy (KPE). We evaluated the predictive nature of pre-KPE clinical and histological factors on transplant-free survival (TFS) and jaundice clearance. Methods: A retrospective review of patients who received a KPE at our institution (1997–2018) was performed. Primary outcomes were two-year TFS, five-year TFS, and jaundice clearance 3 months after KPE. *p* < 0.05 was considered significant. Results: Fifty-four patients were included in this study. The two-year TFS was 35.1%, five-year TFS was 24.5%, and 37% patients reached a direct bilirubin (DB) ≤ 2.0 mg/dL 3 months post KPE. The median age at biopsy was younger in the five-year TFS (39.0 (24.5–55.5) vs. 56.0 days (51.0–67.0), *p* = 0.011). Patients with DB ≤ 1.0 mg/dL 3 months after KPE were statistically younger at biopsy (DB ≤ 1.0 44.0 (26.0–56.0) vs. DB > 1.0 56.0 days (51.0–69.0), *p* = 0.016). Ductal plate malformation was less frequent in the five-year TFS (16/17, 94.1%, vs. 1/17, 5.9%, *p* = 0.037). Portal fibrosis (19/23, 82.6%, vs. 4/23, 17.4%, *p* = 0.028) and acute cholangitis (6/7, 85.7%, vs. 1/7, 14.3%, *p* = 0.047) occurred less frequently in two-year TFS. Conclusion: Older age at biopsy, acute cholangitis, portal fibrosis, and ductal plate malformation were associated with lower native liver survival. Evaluation in a larger study population is needed to validate these results.

## 1. Introduction

Biliary atresia (BA) is characterized by progressive fibrosing and scarring of the bile ducts preventing bile excretion by the liver. If untreated, it leads to the development of biliary cirrhosis and death in infancy [1,2]. The Kasai portoenterostomy (KPE) is the gold standard for the treatment of BA. The success of this procedure is determined by its ability to clear jaundice. The reported jaundice clearance rate is 55–60% [3]. Even with successful bile drainage, half of children with BA will need a liver transplant (LT) by age 2. Infants whose total bilirubin level remains above 2.0 mg/dL 3 months after KPE have been shown to be at risk for early disease progression [4]. 

The standard of care remains to perform a KPE first and a secondary liver transplant (LT) if it fails [5,6]. In the USA, only patients with advanced liver disease at diagnosis are considered for primary LT. Although rarely performed (0.1–11%), it is associated with good survival and outcomes [2,7,8]. Despite this, surgeons are reluctant to perform a primary LT, since it is estimated that approximately one third of all patients with BA may not require a LT. It is currently very difficult to predict which patients will survive long term with their native liver (“success”) from those who will need a LT within the first two years of life (“failure”). Identification of “early failure” patients could lead to avoiding an unnecessary KPE, with earlier listing and LT, decreasing the wait list morbidity and mortality. 

Many groups have tried to identify clinical and histological predictive factors of post-KPE failure [9,10,11,12], but few groups have tried identifying pre-KPE factors that could predict native liver survival after KPE [13]. We aim to confirm the predictive value of those previously reported histology findings and to identify additional predictive histological and clinical factors. 

## 2. Methods

### 2.1. Patient Selection and Data Collection

Institutional Review Board approval was obtained (IRB 2007-12989, study approved 31 August 2018). Since the surgical technique and number of KPEs per year per surgeon have been shown to influence outcomes after KPE, only patients whose KPE was performed by our institution’s pediatric hepatobiliary surgeon (RS) were included (August 1997–December 2018). Fifty patients were excluded because of the following reasons: pre-KPE biopsy slides were not available for review, they underwent a primary LT, they did not have a liver biopsy before the KPE, or they had <2 years followup. Fifty-four patients were ultimately included in this study.

### 2.2. Outcomes and Clinical Factors

Primary outcomes were defined as two-year and five-year transplant free survival (TFS) and clearance of jaundice at 3 months after KPE (direct bilirubin (DB) ≤ 2.0 or ≤1.0 mg/dL). Clinical factors that were available pre-KPE were evaluated: age at diagnosis of jaundice, age at liver biopsy, and DB at the time of liver biopsy. 

### 2.3. Histological Factors

Azarow et al. evaluated the predictive nature of pre-KPE liver biopsy histologic findings on post KPE outcomes [13]. These factors were evaluated, as well as those from the “Histological assessment for cholestasis in infancy” developed by the Biliary Atresia Research Consortium (BARC) [14]. The list of the histologic features that were reviewed is presented in Table 1. 

All 54 pre KPE liver biopsies were reviewed blindly by our institutional pediatric liver pathologist (HMA) and two pediatric hepatobiliary surgeons (CL, RS). Participants were unaware of the patient’s identity and KPE outcome. To reduce interobserver variation, all features were evaluated with a tertiary (0 = absent/mild, 1 = moderate, 2 = severe, or 0 = absent/minimally present, 1 = present, 2 = prominent) and a binary system (0 = absent/mild, 1 = moderate/severe, or 0 = absent/minimally present, 1 = present/prominent), except for the ductal plate malformation (DPM) and bile in zone 1 (binary scoring system only). 

### 2.4. Statistical Analysis

The Chi-square test and Fisher’s exact test were used for univariate analyses of categorical variables, while linear logistic regression was used for continuous variables. Statistics were performed using the IBM SPSS Statistics program (version 24.0.0.0). A *p* < 0.05 was considered statistically significant. A *p* < 0.1 was used to account for the limitations from the small sample size in observing statistical trends. 

## 3. Results

### 3.1. Descriptive Statistics and Outcomes

The patients’ characteristics are presented in Table 2. Fifty-four patients met the inclusion criteria and were included in this study (fifty patients were excluded). The median gestational age was 39 weeks (range 28–42); seven patients were born prematurely. Most patients were diagnosed with jaundice on the first day of life (range 0–78). In 26 patients, an alternative explanation for jaundice was mentioned. Only one patient had a biliary atresia splenic malformation. The predictive value of this factor was therefore not evaluated. The median age at the time of initial hepatology evaluation was 51 days (range 3–81), while the median age at liver biopsy was 55.5 days (range 19–152). The median DB level was 5.3 mg/dL (2.5–14.7). All patients underwent a KPE (median age 59 days (range 22–153)). 

The median DB level 3 months after KPE was 4.1 mg/dL (0.1–13.9). Nineteen patients (37.3%) reached a DB ≤ 2.0 3 months after KPE, while 14 (27.5%) achieved a DB ≤ 1.0. The two-year TFS was 35.2% (19/54). The five-year TFS was 24.5% (12/49). Of note, the 19 patients who reached a DB ≤ 1.0 were not the same as those who had a two-year TFS. 

### 3.2. Univariate Analysis of Clinical and Histological Factors Based on Individual Outcome 

**Figure 1:**
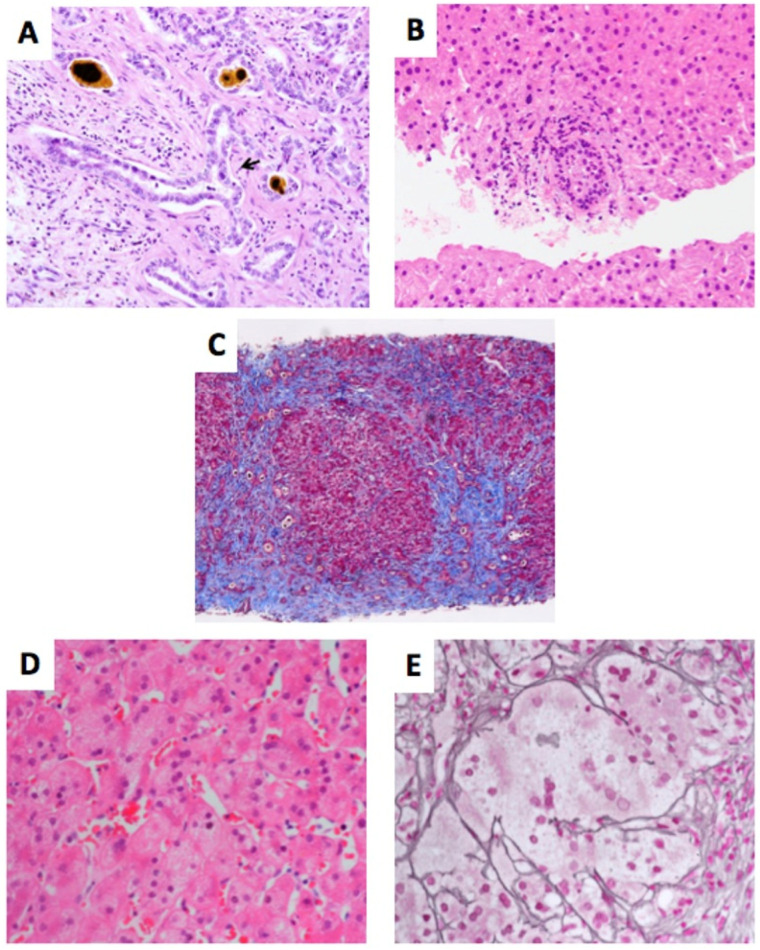
Histology findings found to be statistically significant (*p* < 0.05: (**A**–**C**)) or a statistical trend (*p* < 0.1: (**D**,**E**)) with either TFS or DB level at 3 months after KPE using univariate analysis. (Legend: (**A**): DPM; (**B**): acute cholangitis; (**C**): portal fibrosis; (**D**): multinucleated giant cells; (**E**): syncytial giant cells) ((**A**): Cytokeratin-19 stain; (**B**,**D**): hematoxylin and eosin stain; (**C**): Trichrome Masson stain; (**E**): reticulin stain).

Clinical and histological factors that either reached statistical significance or displayed a trend in predicting any outcome are presented in Table 3 (binary scoring system) and Table 4 (tertiary scoring system). Representative histology slides of these features are shown in Figure 1. Other histology findings that were evaluated in the statistical model are presented in the Supplemental Tables S1 and S2 (Table S1 shows the univariate analysis using the binary scoring system, Table S2 shows the results of the univariate analysis using the tertiary scoring system).

#### 3.2.1. Two-Year Transplant-Free Survival

Fifty-four patients were included in the two-year TFS analysis. There were no clinical factors that could predict the two-year TFS. The median age at the onset of jaundice was similar. The median age at biopsy and median DB at biopsy were higher in the LT group but did not reach statistical significance (55 days (IQR 51.0, 67.0) vs. 48 days (IQR 26.0, 64.0), *p* = 0.14). Using the binary scoring system of the histologic features, none reached statistical significance. The multinucleated giant cells on liver biopsy showed a trend in failure after KPE (76.9% vs. 23.1%, *p* = 0.073). When using the tertiary scoring system, portal fibrosis (82.6% vs. 17.4%, *p* = 0.028) and acute cholangitis (85.7% vs. 14.3%, *p* = 0.047) were found to be statistically significant. Severe portal fibrosis, severe acute cholangitis, and moderate acute cholangitis were more prevalent in the two-year LT group. 

#### 3.2.2. Five-Year Transplant Free Survival

Forty-nine patients were included in the five-year TFS analysis. The median age at biopsy was significantly lower in the five-year TFS group (56 days (IQR 51.0, 67.0) vs. 39 (IQR 24.0, 55.5), *p* = 0.011). DB at the time of liver biopsy was lower in the five-year TFS group, but not significant (0 days (IQR 0.0, 7.0) vs. 10 days (IQR 0.0, 14.5), *p* = 0.63). When evaluating liver biopsies with the binary scoring system, the presence of DPM was associated with failure (94.1% vs. 5.9%, *p* = 0.037). Acute cholangitis showed a trend towards KPE failure (85.2% vs. 14.8%, *p* = 0.081). Using the tertiary scoring system, severe acute cholangitis reached statistical significance for failure (100.0% vs. 0.0%, *p* = 0.038). Severe portal fibrosis was associated with a trend in KPE failure (87.0% vs. 13.0%, *p* = 0.072). 

#### 3.2.3. Clearance of Jaundice Three Months after KPE

When evaluating patients who reached a DB ≤ 2.0 (*n* = 19), only the presence of acute cholangitis on the binary scoring system showed a trend towards failure (74.1% vs. 25.9%, *p* = 0.076). Fourteen patients successfully reached a DB ≤ 1.0 3 months after KPE. They were statistically younger at liver biopsy (44 days (IQR 26.0, 56.0) vs. 56 days (IQR 51.0, 69.0), *p* = 0.016). None of the histologic factors reached statistical significance with the binary scoring system. With the tertiary scoring system, three histologic features showed a trend towards failure: severe portal fibrosis (85.7% vs. 14.3%, *p* = 0.066), severe acute cholangitis (100.0% vs. 0.0%, *p* = 0.063), and numerous syncytial giant cells (100.0% vs. 0.0%, *p* = 0.085). 

## 4. Discussion

While the KPE was a groundbreaking development in the management of infants with BA, for most patients it remains a palliative procedure serving as a bridge to LT [15]. Reported native liver survival in Western countries varies between 20% and 56%, although the length of followup varies greatly, making comparisons difficult. [7] Most of the research aimed at identifying the predictive factors of BA outcomes focused on intra- or postoperative factors. We sought to identify predictive factors of “early failure post-KPE” relying on preoperative clinical and histological factors. Our results suggest that older age at biopsy, DPM, moderate to severe portal fibrosis, and acute cholangitis are predictive factors of failure. Additionally, multinucleated giant cells and syncytial giant cells could be associated with a lower native liver survival. 

Younger age at KPE is frequently cited as a favorable factor to achieve TFS and resolution of jaundice [16,17,18,19]. Patients who receive a KPE at a younger age undergo a diagnostic liver biopsy at a younger age, explaining how the younger age at biopsy was associated with five-year TFS and achieving a DB ≤ 1.0 3 months after KPE in our study. The ability to diagnose BA on early liver biopsy has been questioned, as “typical BA findings” may not be present early or in premature infants [20,21]. However, a recent meta-analysis showed there was no difference in the accuracy of liver biopsy in patients younger or older than 60 days [22]. In our study, nearly half of the patients had an “alternative explanation” for their jaundice. Pediatric gastroenterologists recommend investigating jaundice if it persists 3 weeks after birth [23]. Earlier identification of direct hyperbilirubinemia could lead to earlier liver biopsy and improved post KPE outcomes. Therefore, when a liver biopsy shows features suggestive of BA, an operative cholangiogram should be performed promptly to allow patients to undergo a KPE at the youngest possible age, possibly allowing jaundice clearance and survival with their native liver. 

The presence of moderate or severe acute cholangitis on liver biopsy was the only histologic feature found to be associated with all three outcomes investigated in this study. This is the first report of the predictive nature of acute cholangitis on pre-KPE liver biopsy. Infection and inflammation in already abnormal bile ducts may lead to unrecoverable intrinsic injury despite KPE. In the BARC assessment, interobserver agreement was the poorest in the histologic features of inflammation such as cholangitis [14]. The BARC scoring system uses a 4-tier score. Reducing it to a binary or tertiary scoring system, as we suggest, may improve pathologists’ agreement. 

The presence of fibrosis on liver biopsy is a controversial predictor of post-KPE outcome [24,25]. Studies have reported fibrosis as a predictor of poor outcomes as it could compromise bile outflow [26,27,28,29,30]. Webb et al. reported that the absence of bridging fibrosis on liver biopsy was the only factor significantly associated with improved five-year TFS [31]. Another group showed that high-grade fibrosis was an indicator of poor postoperative prognosis even when KPE was performed in young patients [27]. Our experience is concordant with these groups. 

DPM has been thought to represent an interruption of the normal remodeling process of the biliary tract during fetal life [32]. Its incidence in patients with BA has been reported between 20 and 50% [33]. DPM is considered a marker of antenatal onset of disease leading to a longer duration of liver injury and has been associated with a lower jaundice clearance rate 3 months after KPE and a shorter interval to LT [27,30,33,34,35,36]. Low et al. reported that all their BA patients with DPM on biopsy had a poor outcome [33]. Safwan et al. reported that 69% of patients in their study who underwent a primary LT had evidence of DPM on biopsy, and DPM was associated with a shorter native-liver survival after KPE [35]. In our cohort, thirty-five percent of patients had DPM identified on their pre KPE liver biopsy, and the presence of DPM was associated with a lower five-year TFS. 

Multinucleated hepatocytes are individual hepatocytes containing three or more nuclei [14]. Syncytial giant cells were originally described as large conglomerate of hepatocytes containing up to 30 nuclei [37]. Azarow et al. reported that both were associated with poor KPE outcomes [13]. Vazquez et al. reported the same association but on KPE surgical specimens [38]. A recent study concluded that the presence of hepatitis-like features was an indicator of poor short-term jaundice clearance [39]. These findings correlate with ours, where multinucleated giant cells were associated with a lower two-year TFS, and syncytial giant cells were associated with a lower jaundice clearance post-KPE. 

The authors recognize the limitations of this study. First, it is a retrospective single institution study with a small sample size. This is explained by the exclusion of 50 additional patients due to liver biopsy performed at other institutions being unavailable for review; the absence of a preoperative biopsy; primary LT; and other types of biliary drainage procedure. Second, the liver biopsies were reviewed by a single pathologist. Since this pathologist reviews all liver biopsies performed at our institution, it did not appear helpful to have other pathologists participate in the review, especially since interobserver agreement between expert pediatric liver pathologists can be challenging [14]. 

## 5. Conclusions

In conclusion, a combination of clinical factors (younger age at biopsy) and the presence of histologic factors on the diagnostic liver biopsy (presence of severe acute cholangitis, portal fibrosis, and ductal plate malformation) of infants with BA can likely predict early failure after KPE and would identify patients who could benefit from a primary LT. A positive predictive score with a high degree of sensitivity and specificity that combines elements of both clinical and histological parameters could be used to stream patients at high risk for early failure into a primary transplant arm rather than having them undergo an operation that could be futile and that would complicate any probably future liver transplant. A larger multicenter study will be needed to externally validate the findings of this study. It will also allow the evaluation of other pertinent clinical and histologic factors, as a larger study population may generate stronger results to be incorporated in a predictive score of BA outcomes. 

## Figures and Tables

**Table 1:** List of histologic factors reviewed on liver biopsies as potential predictive factors of outcomes (Legend: BARC: Biliary Atresia Research Consortium).

Histologic Findings		Source
Portal tracts	Portal fibrosis	BARC
	Portal inflammation	BARC
	Portal edema	BARC
	Bile in zone 1	Azarow et al. [13]
Bile ducts	Bile duct proliferation	BARC
	Bile duct damage	BARC
	Acute cholangitis	BARC/Azarow et al. [13]
	Portal ductular reaction	BARC
	Ductal plate malformation	BARC
	Focal necrosis	Azarow et al. [13]
	Bridging necrosis	BARC/Azarow et al. [13]
Hepatocytes	Hepatocellular cholestasis	BARC
	Lobular inflammation	BARC/Azarow et al. [13]
	Multinucleated giant hepatocytes	BARC
	Syncytial giant cells	Azarow et al. [13]
	Individually necrotic hepatocytes	BARC
	Hepatocellular rosettes	BARC

**Table 2:** Descriptive statistics of the study population (Legend: KPE: Kasai portoenterostomy; (‡) One patient was both breastfed and thought to have a perinatal infection; (§) Non-corrected age for prematurely born patients).

Variable	Results
	(*n* = 54)
Sex (*n*, male, %)	26 (48.1)
Gestational age (weeks, median, range)	39 (28–42)
Prematurity (*n*, yes, %)	7 (13.0)
Age at onset of jaundice (days, median, range)	1 (0–78)
Potential explanation for jaundice (*n*, yes, %)	26 (48.1) ^‡^
Breastfeeding	20 (37)
Perinatal infection	5 (9.3)
Galactosemia	1 (1.9)
Total parenteral nutrition	1 (1.9)
ABO incompatibility	0 (0)
Biliary atresia splenic malformation (*n*, yes, %)	1 (1.9)
Age at biopsy (days, median, range) ^§^	55.5 (19.0–152.0)
Aspartate aminotransferase at biopsy (IU/L, median, range)	168.5 (48–451)
Alanine aminotransferase at biopsy (IU/L, median, range)	110 (20–375)
Direct bilirubin at biopsy (mg/dL, median, range)	5.3 (2.5–14.7)
Age at KPE (days, median, range) ^§^	59.0 (22.0–153.0)

**Table 3:** Univariate analysis of clinical and histologic factors, binary scoring system. (Legend: DB: Direct bilirubin; KPE: Kasai portoenterostomy; (*): *p* value < 0.05 statistically significant; (†): *p* value < 0.1, statistical trend).

Variables		2-Year Transplant-Free Survival			5-Year Transplant-Free Survival			DB ≤ 2 at 3 Months after KPE			DB ≤ 1 at 3 Months after KPE		
		No	Yes	*p*-Value	No	Yes	*p*-Value	No	Yes	*p*-Value	No	Yes	*p*-Value
		(*n* = 35)	(*n* = 19)		(*n* = 37)	(*n* = 12)		(*n* = 32)	(*n* = 19)		(*n* = 37)	(*n* = 14)	
Age at biopsy (days, median, IQR)		55.0 (51.0, 67.0)	48.0 (26.0, 64.0)	0.14	56.0 (51.0, 67.0)	39.0 (24.0, 55.5)	0.011 *	55.0 (45.5, 68.0)	55.0 (29.0, 63.0)	0.19	56.0 (51.0, 69.0)	44.0 (26.0, 56.0)	0.016 *
Ductal plate malformation (*n*, %)	0	20 (57.1)	15 (42.9)	0.11	21 (65.6)	11 (34.4)	0.037 *	20 (58.8)	14 (41.2)	0.41	23 (67.6)	11 (32.4)	0.33
	1	15 (78.9)	4 (21.1)		16 (94.1)	1 (5.9)		12 (70.6)	5 (29.4)		14 (82.4)	3 (17.6)	
Acute cholangitis (*n*, %)	0	14 (53.8)	12 (46.2)	0.10	14 (63.6)	8 (36.4)	0.081 ^†^	12 (50.0)	12 (50.0)	0.076 ^†^	15 (62.5)	9 (37.5)	0.13
	1	21 (75.0)	7 (25.0)		23 (85.2)	4 (14.8)		20 (74.1)	7 (25.9)		22 (81.5)	5 (18.5)	
Multinucleated giant cells (*n*, %)	0	15 (53.6)	13 (46.4)	0.073 ^†^	16 (66.7)	8 (33.3)	0.16	16 (61.5)	10 (38.5)	0.86	17 (65.4)	9 (34.6)	0.24
	1	20 (76.9)	6 (23.1)		21 (84.0)	4 (16.0)		16 (64.0)	9 (36.0)		20 (80.0)	5 (20.0)	

**Table 4:** Univariate analysis of clinical and histologic factors, tertiary scoring system. (Legend: DB: Direct bilirubin; KPE: Kasai portoenterostomy; (*): *p* value < 0.05 statistically significant; (†): *p* value < 0.1, statistical trend).

Variables		2-Year Transplant-Free Survival			5-Year-Transplant Free Survival			DB ≤ 2 at 3 Months after KPE			DB ≤ 1 at 3 Months after KPE		
		No	Yes	*p*-Value	No	Yes	*p*-Value	No	Yes	*p*-Value	No	Yes	*p*-Value
		(*n* = 35)	(*n* = 19)		(*n* = 37)	(*n* = 12)		(*n* = 32)	(*n* = 19)		(*n* = 37)	(*n* = 14)	
Age at biopsy (days, median, IQR)		55.0 (51.0, 67.0)	48.0 (26.0, 64.0)	0.14	56.0 (51.0, 67.0)	39.0 (24.0, 55.5)	0.011 *	55.0 (45.5, 68.0)	55.0 (29.0, 63.0)	0.19	56.0 (51.0, 69.0)	44.0 (26.0, 56.0)	0.016 *
Portal fibrosis (*n*, %)	0	1 (25.0)	3 (75.0)	0.028 *	1 (33.3)	2 (66.7)	0.072 ^†^	2 (40.0)	3 (60.0)	0.40	2 (40.0)	3 (60.0)	0.066 ^†^
	1	15 (55.6)	12 (44.4)		16 (69.6)	7 (30.4)		15 (60.0)	10 (40.0)		17 (68.0)	8 (32.0)	
	2	19 (82.6)	4 (17.4)		20 (87.0)	3 (13.0)		15 (71.4)	6 (28.6)		18 (85.7)	3 (14.3)	
Acute cholangitis (*n*, %)	0	10 (45.5)	12 (54.5)	0.047 *	10 (55.6)	8 (44.4)	0.038 *	11 (52.4)	10 (47.6)	0.30	12 (57.1)	9 (42.9)	0.063 ^†^
	1	19 (76.0)	6 (24.0)		20 (83.3)	4 (16.7)		15 (65.2)	8 (34.8)		18 (78.3)	5 (21.7)	
	2	6 (85.7)	1 (14.3)		7 (100)	0 (0.0)		6 (85.7)	1 (14.3)		7 (100)	0 (0.0)	
Syncytial giant cells (*n*, %)	0	16 (55.2)	13 (44.8)	0.28	17 (68.0)	8 (32.0)	0.21	15 (57.7)	11 (42.3)	0.29	16 (61.5)	10 (38.5)	0.085 ^†^
	1	12 (75.0)	4 (25.0)		12 (75.0)	4 (25.0)		10 (58.8)	7 (41.2)		13 (76.5)	4 (23.5)	
	2	7 (77.8)	2 (22.2)		8 (100)	0 (0.0)		7 (87.5)	1 (12.5)		8 (100)	0 (0.0)	

## Data Availability

The data that support the findings of this study are available from the corresponding author upon reasonable request.

## Supplementary Materials

The following supporting information can be downloaded at: https://www.mdpi.com/article/10.3390/jcm11216523/s1, Table S1: Additional data to Table 3: Univariate analysis of clinical and histologic factors using the binary scoring system; Table S2: Additional data to Table 4: Univariate analysis of clinical and histologic factors using the tertiary scoring system.

## Abbreviations

BA	Biliary atresia
BARC	Biliary atresia research consortium
DB	Direct bilirubin
KPE	Kasai portoenterostomy
LT	Liver transplantation
TB	Total bilirubin
TFS	Transplant-free survival

## References

[1] Karrer F.M., Price M.R., Bensard D.D., Sokol R.J., Narkewicz M.R., Smith D.J., Lilly J.R. (1996). Long-term results with the Kasai operation for biliary atresia. Arch. Surg..

[2] Superina R. (2013). Liver transplantation for biliary atresia: Does the insurance type really make a difference?. Liver Transplant..

[3] Lakshminarayanan B., Davenport M. (2016). Biliary atresia: A comprehensive review. J. Autoimmun..

[4] Shneider B.L., Magee J.C., Karpen S.J., Rand E.B., Narkewicz M.R., Bass L.M., Schwarz K., Whitington P.F., Bezerra J.A., Kerkar N. (2016). Total Serum Bilirubin within 3 Months of Hepatoportoenterostomy Predicts Short-Term Outcomes in Biliary Atresia. J. Pediatr..

[5] Davenport M., Goyet J.D.V.D., Stringer M., Mieli-Vergani G., Kelly D., McClean P., Spitz L. (2004). Seamless management of biliary atresia in England and Wales (1999–2002). Lancet.

[6] Otte J.-B., Goyet J.D.V.D., Reding R., Hausleithner V., Sokal E., Chardot C., Debande B. (1994). Sequential treatment of biliary atresia with kasai portoenterostomy and liver transplantation: A review. Hepatology.

[7] Superina R. (2017). Biliary atresia and liver transplantation: Results and thoughts for primary liver transplantation in select patients. Pediatr. Surg. Int..

[8] Wang P., Xun P., He K., Cai W. (2016). Comparison of liver transplantation outcomes in biliary atresia patients with and without prior portoenterostomy: A meta-analysis. Dig. Liver Dis..

[9] Chen G., Zheng S., Sun S., Xiao X., Ma Y., Shen W., Chen L., Song Z. (2012). Early surgical outcomes and pathological scoring values of older infants (≥90 d old) with biliary atresia. J. Pediatr. Surg..

[10] Sun S., Zheng S., Lu X., Chen G., Ma Y., Chen L., Dong K. (2018). Clinical and pathological features of patients with biliary atresia who survived for more than 5 years with native liver. Pediatr. Surg. Int..

[11] Goda T., Kawahara H., Kubota A., Hirano K., Umeda S., Tani G., Ishii T., Tazuke Y., Yoneda A., Etani Y. (2013). The most reliable early predictors of outcome in patients with biliary atresia after Kasai’s operation. J. Pediatr. Surg..

[12] Ihn K., Ho I.G., Chang E.Y., Han S.J. (2018). Correlation between gamma-glutamyl transpeptidase activity and outcomes after Kasai portoenterostomy for biliary atresia. J. Pediatr. Surg..

[13] Azarow K.S., Phillips M.J., Sandler A.D., Hagerstrand I., Superina R.A. (1997). Biliary atresia: Should all patients undergo a portoenterostomy?. J. Pediatr. Surg..

[14] Russo P., Magee J.C., Boitnott J., Bove K.E., Raghunathan T., Finegold M., Haas J., Jaffe R., Kim G.E., Magid M. (2011). Design and Validation of the Biliary Atresia Research Consortium Histologic Assessment System for Cholestasis in Infancy. Clin. Gastroenterol. Hepatol..

[15] Hartley J.L., Davenport M., Kelly D.A. (2009). Biliary atresia. Lancet.

[16] Superina R., Magee J.C., Brandt M.L., Healey P.J., Tiao G., Ryckman F., Karrer F.M., Iyer K., Fecteau A., West K. (2011). The Anatomic Pattern of Biliary Atresia Identified at Time of Kasai Hepatoportoenterostomy and Early Postoperative Clearance of Jaundice Are Significant Predictors of Transplant-Free Survival. Ann. Surg..

[17] Lien T.-H., Chang M.-H., Wu J.-F., Chen H.-L., Lee H.-C., Chen A.-C., Tiao M.-M., Wu T.-C., Yang Y.-J., Lin C.-C. (2011). Effects of the infant stool color card screening program on 5-year outcome of biliary atresia in taiwan. Hepatology.

[18] Schreiber R.A., Barker C.C., Roberts E.A., Martin S.R., Alvarez F., Smith L., Butzner J.D., Wrobel I., Mack D., Moroz S. (2007). Biliary Atresia: The Canadian Experience. J. Pediatr..

[19] Serinet M.-O., Wildhaber B.E., Broué P., Lachaux A., Sarles J., Jacquemin E., Gauthier F., Chardot C. (2009). Impact of Age at Kasai Operation on Its Results in Late Childhood and Adolescence: A Rational Basis for Biliary Atresia Screening. Pediatrics.

[20] Ferry G.D., Selby M.L., Udall J., Finegold M., Nichols B. (1985). Guide to Early Diagnosis of Biliary Obstruction in Infancy. Clin. Pediatr..

[21] Mowat A.P., Psacharopoulos H.T., Williams R. (1976). Extrahepatic biliary atresia versus neonatal hepatitis. Review of 137 prospectively investigated infants. Arch. Dis. Child..

[22] Lee J.Y., Sullivan K., El Demellawy D., Nasr A. (2016). The value of preoperative liver biopsy in the diagnosis of extrahepatic biliary atresia: A systematic review and meta-analysis. J. Pediatr. Surg..

[23] Fawaz R., Baumann U., Ekong U., Fischler B., Hadzic N., Mack C.L., McLin V.A., Molleston J.P., Neimark E., Ng V.L. (2017). Guideline for the Evaluation of Cholestatic Jaundice in Infants: Joint Recommendations of the North American Society for Pediatric Gastroenterology, Hepatology, and Nutrition and the European Society for Pediatric Gastroenterology, Hepatology, and Nutrition. J. Pediatr. Gastroenterol. Nutr..

[24] Bhatnagar V., Agarwala S., Gupta S.D., Baruah R.R. (2015). Correlation of pre- and post-operative liver function, duct diameter at porta hepatis, and portal fibrosis with surgical outcomes in biliary atresia. J. Indian Assoc. Pediatr. Surg..

[25] Czubkowski P., Cielecka-Kuszyk J., Rurarz M., Kaminska D., Markiewicz-Kijewska M., Pawlowska J. (2015). The limited prognostic value of liver histology in children with biliary atresia. Ann. Hepatol..

[26] Weerasooriya V.S., White F.V., Shepherd R. (2004). Hepatic fibrosis and survival in biliary atresia. J. Pediatr..

[27] Muthukanagarajan S.J. (2016). Diagnostic and Prognostic Significance of Various Histopathological Features in Extrahepatic Biliary Atresia. J. Clin. Diagn. Res..

[28] Arii R., Koga H., Arakawa A., Miyahara K., Lane G.J., Okazaki T., Urao M., Yamataka A. (2010). How valuable is ductal plate malformation as a predictor of clinical course in postoperative biliary atresia patients?. Pediatr. Surg. Int..

[29] Salzedas-Netto A., Chinen E., de Oliveira D., Pasquetti A., Azevedo R., Patricio F.D.S., Cury E., Gonzalez A., Vicentine F., Martins J. (2014). Grade IV Fibrosis Interferes in Biliary Drainage After Kasai Procedure. Transplant. Proc..

[30] Russo P., Magee J.C., Anders R.A., Bove K.E., Chung C., Cummings O.W., Finegold M.J., Finn L.S., Kim G.E., Lovell M.A. (2016). Key Histopathologic Features of Liver Biopsies That Distinguish Biliary Atresia From Other Causes of Infantile Cholestasis and Their Correlation With Outcome. Am. J. Surg. Pathol..

[31] Webb N.L., Jiwane A., Ooi C., Nightinghale S., Adams S.E., Krishnan U. (2017). Clinical significance of liver histology on outcomes in biliary atresia. J. Paediatr. Child Health.

[32] Raynaud P., Tate J., Callens C., Cordi S., Vandersmissen P., Carpentier R., Sempoux C., Devuyst O., Pierreux C.E., Courtoy P. (2011). A classification of ductal plate malformations based on distinct pathogenic mechanisms of biliary dysmorphogenesis. Hepatology.

[33] Low Y., Vijayan V., Tan C.E. (2001). The prognostic value of ductal plate malformation and other histologic parameters in biliary atresia: An immunohistochemical study. J. Pediatr..

[34] Shimadera S., Iwai N., Deguchi E., Kimura O., Ono S., Fumino S., Higuchi K. (2008). Significance of ductal plate malformation in the postoperative clinical course of biliary atresia. J. Pediatr. Surg..

[35] Safwan M., Ramachandran P., Vij M., Shanmugam N., Rela M. (2015). Impact of ductal plate malformation on survival with native liver in children with biliary atresia. Pediatr. Surg. Int..

[36] Roy P., Chatterjee U., Ganguli M., Banerjee S., Chatterjee S., Basu A. (2010). A histopathological study of liver and biliary remnants with clinical outcome in cases of extrahepatic biliary atresia. Indian J. Pathol. Microbiol..

[37] Phillips M.J., Blendis L.M., Poucell S., Patterson J., Petric M., Roberts E., Levy G.A., Superina R.A., Greig P.D., Cameron R. (1991). Syncytial Giant-Cell Hepatitis. N. Engl. J. Med..

[38] Vazquez-Estevez J., Stewart B., Shikes R.H., Hall R.J., Lilly J.R. (1989). Biliary atresia: Early determination of prognosis. J. Pediatr. Surg..

[39] Suda K., Muraji T., Ohtani H., Aiyoshi T., Sasaki T., Toma M., Yanai T. (2019). Histological significance of hepatitis-like findings in biliary atresia: An analysis of 34 Japanese cases. Pediatr. Int..

