# Supplementary material for: Identification of Early Clinical and Histological Factors Predictive of Kasai Portoenterostomy Failure
Source: J Clin Med. 2022 Nov 3;11(21):6523. doi: 10.3390/jcm11216523 (PMC9654517; doi:10.3390/jcm11216523)
Supplement: Supplementary file 1 [file jcm-11-06523-s001.zip › jcm-1870097-supplementary.pdf]

Supplemental Table S1. Additional data to Table 3: Univariate analysis of clinical and histologic factors using the binary scoring system.

Variables		2 Year Transplant Free Survival			5 Year Transplant Free Survival			Bilirubin ≤ 2 at 3 months after KPE			Bilirubin ≤ 1 at 3 months after KPE		
		No (n=35)	Yes (n=19)	P-value	No (n=37)	Yes (n=12)	P-value	No (n=32)	Yes (n=19)	P-value	No (n=37)	Yes (n=14)	P-value
Age at onset of jaundice (days, median, IQR)		0.0 (0.0, 3.0)	3.0 (0.0, 14.0)	0.48	0.0 (0.0, 7.0)	10.0 (0.0, 14.5)	0.63	5.4 (4.3, 6.5)	5.1 (4.3, 5.9)	0.27	5.5 (4.4, 6.7)	4.8 (3.8, 5.9)	0.23
Direct bilirubin at GI evaluation (mg/dL, median, IQR)		5.7 (4.7, 7.3)	4.8 (3.8, 5.9)	0.12	5.5 (5.0, 7.2)	4.7 (3.8, 6.0)	0.17	0.0 (0.0, 3.0)	3.0 (0.0, 14.0)	0.37	0.0 (0.0, 3.0)	4.5 (0.0, 14.0)	0.62
Portal fibrosis	0	3 (37.5)	5 (62.5)	0.11	3 (60.0)	2 (40.0)	0.58	5 (62.5)	3 (37.5)	1.00	5 (62.5)	3 (37.5)	0.67
	1	32 (69.6)	14 (30.4)		34 (77.3)	10 (22.7)		27 (62.8)	16 (37.2)		32 (74.4)	11 (25.6)	
Portal ductular reaction	0	9 (56.3)	7 (43.8)	0.39	9 (60.0)	6 (40.0)	0.15	7 (46.7)	8 (53.3)	0.13	9 (60.0)	6 (40.0)	0.30
	1	26 (68.4)	12 (31.6)		28 (82.4)	6 (17.6)		25 (69.4)	11 (30.6)		28 (77.8)	8 (22.2)	
Hepatocellular cholestasis	0	35 (67.3)	17 (32.7)	0.12	36 (76.6)	11 (23.4)	0.43	32 (65.3)	17 (34.7)	0.13	36 (73.5)	13 (26.5)	0.48
	1	0 (0.0)	2 (100)		1 (50.0)	1 (50.0)		0 (0.0)	2 (100)		1 (50.0)	1 (50.0)	
Bile duct proliferation	0	3 (75.0)	1 (25.0)	1.0	3 (100)	0 (0.0)	0.57	4 (100)	0 (0.0)	0.28	4 (100)	0 (0.0)	0.56
	1	32 (64.0)	18 (36.0)		34 (73.9)	12 (26.1)		28 (59.6)	19 (40.4)		33 (70.2)	14 (29.8)	
Portal inflammation	0	7 (58.3)	5 (41.7)	0.73	7 (63.6)	4 (36.4)	0.43	6 (54.5)	5 (45.5)	0.73	7 (63.6)	4 (36.4)	0.47
	1	28 (66.7)	14 (33.3)		30 (78.9)	8 (21.1)		26 (65.0)	14 (35.0)		30 (75.0)	10 (25.0)	
Portal edema	0	13 (56.5)	10 (43.5)	0.27	13 (68.4)	6 (31.6)	0.50	16 (69.6)	7 (30.4)	0.36	17 (73.9)	6 (26.1)	0.84
	1	22 (71.0)	9 (29.0)		24 (80.0)	6 (20.0)		16 (57.1)	12 (42.9)		20 (71.4)	8 (28.6)	
Bile duct injury	0	15 (57.7)	11 (42.3)	0.29	16 (69.6)	7 (30.4)	0.36	14 (60.9)	9 (39.1)	0.80	16 (69.6)	7 (30.4)	0.67
	1	20 (71.4)	8 (28.6)		21 (80.8)	5 (19.2)		18 (64.3)	10 (35.7)		21 (75.0)	7 (25.0)	
Bile in zone 1	0	28 (63.6)	16 (36.4)	1.00	30 (76.9)	9 (23.1)	0.69	28 (63.6)	16 (36.4)	1.00	32 (72.7)	12 (27.3)	1.00
	1	7 (70.0)	3 (30.0)		7 (70.0)	3 (30.0)		4 (57.1)	3 (42.9)		5 (71.4)	2 (28.6)	
Lobular inflammation	0	27 (64.3)	15 (35.7)	1.00	28 (73.7)	10 (26.3)	0.71	23 (59.0)	16 (41.0)	0.50	28 (71.8)	11 (28.2)	1.00

	1	8 (66.7)	4 (33.3)		9 (81.8)	2 (18.2)		9 (75.0)	3 (25.0)		9 (75.0)	3 (25.0)	
Hepatocellular rosettes	0	25 (61.0)	16 (39.0)	0.34	26 (72.2)	10 (27.8)	0.47	24 (61.5)	15 (38.5)	1.00	27 (69.2)	12 (30.8)	0.47
	1	10 (76.9)	3 (23.1)		11 (84.6)	2 (15.4)		8 (66.7)	4 (33.3)		10 (83.3)	2 (16.7)	
Syncitial giant cells	0	22 (61.1)	14 (38.9)	0.42	23 (71.9)	9 (28.1)	0.50	21 (63.6)	12 (36.4)	0.86	22 (66.7)	11 (33.3)	0.33
	1	13 (72.2)	5 (27.8)		14 (82.4)	3 (17.6)		11 (61.1)	7 (38.9)		15 (83.3)	3 (16.7)	
Bridging necrosis	0	29 (65.9)	15 (34.1)	0.73	30 (76.9)	9 (23.1)	0.69	26 (63.4)	15 (36.6)	1.00	30 (73.2)	11 (26.8)	1.00
	1	6 (60.0)	4 (40.0)		7 (70.0)	3 (30.0)		6 (60.0)	4 (40.0)		7 (70.0)	3 (30.0)	
Focal necrosis	0	26 (63.4)	15 (36.6)	1.00	27 (75.0)	9 (25.0)	1.00	22 (57.9)	16 (42.1)	0.32	26 (68.4)	12 (31.6)	0.47
	1	9 (69.2)	4 (30.8)		10 (76.9)	3 (23.1)		10 (76.9)	3 (23.1)		11 (84.6)	2 (15.4)	
Necrotic hepatocytes	0	31 (67.4)	15 (32.6)	0.43	31 (75.6)	10 (24.4)	1.00	27 (61.4)	17 (38.6)	0.70	31 (70.5)	13 (29.5)	0.66
	1	4 (50.0)	4 (50.0)		6 (75.0)	2 (25.0)		5 (71.4)	2 (28.6)		6 (85.7)	1 (14.3)	
Bile plugs	0	10 (55.6)	8 (44.4)	0.31	10 (66.7)	5 (33.3)	0.47	10 (62.5)	6 (37.5)	0.98	11 (68.8)	5 (31.3)	0.74
	1	25 (69.4)	11 (30.6)		27 (79.4)	7 (20.6)		22 (62.9)	13 (37.1)		26 (74.3)	9 (25.7)	
Gallbladder on ultrasound	0	12 (63.2)	7 (36.8)	0.80	13 (72.2)	5 (27.8)	0.74	11 (64.7)	6 (35.3)	0.88	12 (70.6)	5 (29.4)	1.00
	1	22 (66.7)	11 (33.3)		23 (76.7)	7 (23.3)		20 (62.5)	12 (37.5)		23 (71.9)	9 (28.1)	

Supplemental Table S2. Additional data to Table 4: Univariate analysis of clinical and histologic factors using the tertiary scoring system.

Variables		2 Year Transplant Free Survival			5 Year Transplant Free Survival			Bilirubin ≤ 2 at 3 months after KPE			Bilirubin ≤ 1 at 3 months after KPE		
		No (n=35)	Yes (n=19)	p-value	No (n=37)	Yes (n=12)	p-value	No (n=32)	Yes (n=19)	p-value	No (n=37)	Yes (n=14)	p-value
Age at onset of jaundice (days, median, IQR)		0.0 (0.0, 3.0)	3.0 (0.0, 14.0)	0.48	0.0 (0.0, 7.0)	10.0 (0.0, 14.5)	0.63	5.4 (4.3, 6.5)	5.1 (4.3, 5.9)	0.27	5.5 (4.4, 6.7)	4.8 (3.8, 5.9)	0.23
Direct bilirubin at GI evaluation (mg/dL, median, IQR)		5.7 (4.7, 7.3)	4.8 (3.8, 5.9)	0.12	5.5 (5.0, 7.2)	4.7 (3.8, 6.0)	0.17	0.0 (0.0, 3.0)	3.0 (0.0, 14.0)	0.37	0.0 (0.0, 3.0)	4.5 (0.0, 14.0)	0.62
Bile duct proliferation	0	1 (50.0)	1 (50.0)		1 (100)	0 (0.0)		2 (100)	0 (0.0)		2 (100)	0 (0.0)	
	1	11 (52.4)	10 (47.6)	0.25	12 (63.2)	7 (36.8)	0.31	13 (68.4)	6 (31.6)	0.52	13 (68.4)	6 (31.6)	0.87
	2	23 (74.2)	8 (25.8)		24 (82.8)	5 (17.2)		17 (56.7)	13 (43.3)		22 (73.3)	8 (26.7)	
Portal ductular reaction	0	6 (54.5)	5 (45.5)		6 (60.0)	4 (40.0)		5 (45.5)	6 (54.5)		7 (63.6)	4 (36.4)	
	1	13 (59.1)	9 (40.9)	0.36	14 (70.0)	6 (30.0)	0.19	14 (73.7)	5 (26.3)	0.30	14 (73.7)	5 (26.3)	0.74
	2	16 (76.2)	5 (23.8)		17 (89.5)	2 (10.5)		13 (61.9)	8 (38.1)		16 (76.2)	5 (23.8)	
Portal inflammation	0	3 (60.0)	2 (40.0)		3 (75.0)	1 (25.0)		2 (50.0)	2 (50.0)		3 (75.0)	1 (25.0)	
	1	26 (68.4)	12 (31.6)	0.66	27 (75.0)	9 (25.0)	1.00	24 (68.6)	11 (31.4)	0.45	27 (77.1)	8 (22.9)	0.47
	2	6 (54.5)	5 (45.5)		7 (77.8)	2 (22.2)		6 (50.0)	6 (50.0)		7 (58.3)	5 (41.7)	
Portal edema	0	9 (56.3)	7 (43.8)		9 (64.3)	5 (35.7)		10 (62.5)	6 (37.5)		11 (68.8)	5 (31.3)	
	1	14 (66.7)	7 (33.3)	0.67	14 (77.8)	4 (22.2)	0.55	12 (63.2)	7 (36.8)	1.00	13 (68.4)	6 (31.6)	0.73
	2	12 (70.6)	5 (29.4)		14 (82.4)	3 (17.6)		10 (62.5)	6 (37.5)		13 (81.3)	3 (18.8)	
Bile duct injury	0	10 (55.6)	8 (44.4)		11 (73.3)	4 (26.7)		9 (60.0)	6 (40.0)		11 (73.3)	4 (26.7)	
	1	17 (68.0)	8 (32.0)	0.58	17 (70.8)	7 (29.2)	0.62	17 (68.0)	8 (32.0)	0.75	18 (72.0)	7 (28.0)	1.00
	2	8 (72.7)	3 (27.3)		9 (90.0)	1 (10.0)		6 (54.5)	5 (45.5)		8 (72.7)	3 (27.3)	
Lobular inflammation	0	22 (59.5)	15 (40.5)		23 (69.7)	10 (30.3)		20 (58.8)	14 (41.2)		23 (67.6)	11 (32.4)	
	1	12 (80.0)	3 (20.0)	0.30	13 (92.9)	1 (7.1)	0.12	11 (73.3)	4 (26.7)	0.59	13 (86.7)	2 (13.3)	0.28
	2	1 (50.0)	1 (50.0)		1 (50.0)	1 (50.0)		1 (50.0)	1 (50.0)		1 (50.0)	1 (50.0)	
Multinucleated giant cells	0	10 (52.6)	9 (47.4)		11 (64.7)	6 (35.3)		11 (61.1)	7 (38.9)		12 (66.7)	6 (33.3)	
	1	13 (72.2)	5 (27.8)	0.38	13 (81.3)	3 (18.8)	0.53	12 (70.6)	5 (29.4)	0.69	13 (76.5)	4 (23.5)	0.79
	2	12 (70.6)	5 (29.4)		13 (81.3)	3 (18.8)		9 (56.3)	7 (43.8)		12 (75.0)	4 (25.0)	
Hepatocellular rosettes	0	25 (61.0)	16 (39.0)		26 (72.2)	10 (27.8)		24 (61.5)	15 (38.5)		27 (69.2)	12 (30.8)	
	1	6 (75.0)	2 (25.0)	0.70	7 (87.5)	1 (12.5)	0.86	4 (57.1)	3 (42.9)	0.78	6 (85.7)	1 (14.3)	0.86
	2	4 (80.0)	1 (20.0)		4 (80.0)	1 (20.0)		4 (80.0)	1 (20.0)		4 (80.0)	1 (20.0)	
Bridging necrosis	0	29 (65.9)	15 (34.1)	0.75	30 (76.9)	9 (23.1)	0.22	26 (63.4)	15 (36.6)	1.00	30 (73.2)	11 (26.8)	0.40

	1	3 (50.0)	3 (50.0)		3 (50.0)	3 (50.0)		4 (57.1)	3 (42.9)		4 (57.1)	3 (42.9)	
	2	3 (75.0)	1 (25.0)		4 (100)	0 (0.0)		2 (66.7)	1 (33.3)		3 (100)	0 (0.0)	
Necrotic hepatocytes	0	27 (64.3)	15 (35.7)	1.00	27 (73.0)	10 (27.0)	0.78	24 (60.0)	16 (40.0)	0.83	27 (67.5)	13 (32.5)	0.45
	1	7 (63.6)	4 (36.4)		9 (81.8)	2 (18.2)		7 (70.0)	3 (30.0)		9 (90.0)	1 (10.0)	
	2	1 (100)	0 (0.0)		1 (100)	0 (0.0)		1 (100)	0 (0.0)		1 (100)	0 (0.0)	
Hepatocellular cholestasis	0	30 (63.8)	17 (36.2)	0.34	31 (73.8)	11 (26.2)	0.10	29 (64.4)	16 (35.6)	0.60	32 (71.1)	13 (28.9)	0.10
	1	5 (83.3)	1 (16.7)		6 (100)	0 (0.0)		3 (60.0)	2 (40.0)		5 (100)	0 (0.0)	
	2	0 (0.0)	1 (100)		0 (0.0)	1 (100)		0 (0.0)	1 (100)		0 (0.0)	1 (100)	
Focal necrosis	0	22 (59.5)	15 (40.5)	0.57	23 (71.9)	9 (28.1)	0.67	19 (54.3)	16 (45.7)	0.16	23 (65.7)	12 (34.3)	0.26
	1	10 (76.9)	3 (23.1)		10 (76.9)	3 (23.1)		10 (83.3)	2 (16.7)		10 (83.3)	2 (16.7)	
	2	3 (75.0)	1 (25.0)		4 (100)	0 (0.0)		3 (75.0)	1 (25.0)		4 (100)	0 (0.0)	
